# Delusional Parasitosis in a Female Treated with Mixed Amphetamine Salts: A Case Report and Literature Review

**DOI:** 10.1155/2012/624235

**Published:** 2012-09-05

**Authors:** Martha Buscarino, Jaime Saal, Joel L. Young

**Affiliations:** ^1^University of Michigan, Ann Arbor, MI 48109, USA; ^2^Rochester Center for Behavioral Medicine, 441 South Livernois Road, Suite 205, Rochester Hills, MI 48307, USA

## Abstract

*Objectives*. To explore factors underlying the onset of delusional parasitosis; a condition in which an individual has a fixed, false belief that he/she is infested with insects. *Case Description*. MJ is a 57-year-old female who presents with symptoms of fatigue and AD/HD. Upon treatment with extended release mixed amphetamine salts, the patient displayed symptoms of delusional parasitosis. After eventual discontinuation of this medication, her delusions resolved. *Comments*. In order to maintain confidentiality, all identifying information was removed. To this end, please note that MJ is a fictitious name.

## 1. Introduction

Delusional parasitosis is an uncommon psychiatric condition. It is characterized by a patient's fixed, false belief that he/she is infested with insects. This delusion persists despite an absence of medical evidence for infestation. The development of delusional parasitosis may be idiopathic or associated with exposure to various medications. The onset of the condition is multifactorial including the presence of psychiatric or neurological illness. Recent literature emphasizes the use of atypical antipsychotic agents. This study reports the development of transient symptoms of delusional parasitosis after exposure to high levels of extended release mixed amphetamine salts. The symptoms quickly resolved after discontinuation of the offending agent with no additional treatment required. 

## 2. Case Presentation

MJ is a 57-year-old married Caucasian female who initially presented with chronic fatigue. In addition, she reported complaints of irritability, anxiety, and disturbed sleep. Upon further evaluation, the patient endorsed symptoms of generalized pain, impaired concentration, and motivation. She emphasized chronic disorganization, forgetfulness, low energy, and a subjective inability to initiate and complete daily tasks. 

Although her complaints of fatigue were longstanding, MJ's treatment began five years earlier. Having worked with various primary care doctors and psychiatrists, she was frustrated that trials of antidepressant medications, including escitalopram and fluoxetine, had not been helpful. Augmentation with bupropion and risperidone had also proven ineffective. At the time of evaluation she was taking duloxetine 60 mg per day. Various efforts at physical therapy and chiropractic care did not yield lasting benefit. 

MJ had previously pursued specific evaluation for chronic fatigue. Several years ago she was diagnosed with Epstein Barr virus. She was treated with intravenous antiviral agents but derived no more than a transient response. At her current presentation, MJ was being treated with levothyroxine, although her TSH was never detected to be abnormal. She suffers from mild gastroesophageal reflux disease for which she is prescribed omeprazole.

After an extensive psychiatric interview, various diagnostic screeners were administered. These included the Hamilton Depression Inventory [1], the Conners Adult ADHD Rating Scale (CAARS) [2], Millon Clinical Multiaxial Inventory-III [3], the Dementia Rating Scale [4], Mood Disorder Questionnaire [5], and the Adult Self-Report Inventory (ASRI) [6].

The scores revealed significantly elevated scores for ADHD (combined type), moderate levels of depression, and severe generalized anxiety. There was no evidence of dementia, mania, or substance misuse. 

Extended release-mixed amphetamine salts, a first-line stimulant treatment for ADHD, were added to duloxetine. In standard fashion, the mixed amphetamine salts were titrated up to 30 mg/day over the first three weeks. Cognitive-behavioral therapy to address phobic avoidances was recommended but not pursued.

MJ contacted the office within one week and reported improvement in her fatigue and mood. Two weeks later she returned and reported that, in addition to improvements in affective symptoms, she reported greater productivity at work and at home. The medication combination was maintained, and she returned six weeks later. At that visit, MJ and her husband enthusiastically reported less fatigue, improved energy, diminished irritability, and restoration of her sleep-wake cycle. There was no evidence of mania. 

MJ returned one month later. Her improvement persisted, but she reported that 7 hours after her 8 am morning dose of mixed amphetamine salts her symptoms of fatigue and anxiety returned. A second dose of mixed amphetamine salts 20 mg was initiated at 2 PM.

One month later, MJ's husband contacted the clinic and reported that she had deteriorated. In the previous month, she suspected bed bugs in their condominium. In the past ten days, she complained of becoming infested with insects. Her husband denied that this was a possibility. Against his protestations, she began examining the hallways of her ceramic foyer and interpreted small specks on the floor as evidence of fecal remnants. On psychiatric examination, she verified this belief and reported that she had moved out of the apartment and contacted city officials to condemn her luxury condominium. She suspected a conspiracy among the building supervisors, building inspectors, and her husband to minimize the extent of the infestation. She reported her intention to divorce her husband. 

The onset of symptoms was temporally correlated with the increase of the stimulant dosage. For this reason, the mixed amphetamine salts were immediately discontinued. No other psychotropic agents were administered. MJ objected to this plan, fearful that the significant progress she had made on her fatigue and cognitive symptoms would be reversed.

Within two days of discontinuation, the concerns of delusional parasitosis diminished. One week later, MJ completely stopped discussing the delusion. When asked by the physician, she minimized the extent of the delusional parasitosis and concluded that it fundamentally resulted from a vision problem and her husband's exaggeration. On reevaluation one week later, the patient avoided the subject of delusional parasitosis inferring that she found the topic to be unpleasant. She reported that she missed the stimulant medication. Her fatigue and hopelessness had returned; she was unable to awake in the morning and noticed significant productivity loss at work. One month later, after MJ's strong persuasion and the absence of any delusional activity, the mixed amphetamine salts-XR was reintroduced at 20 mg/per day. MJ again derived immediate relief from her incapacitating fatigue. Unfortunately, at the lower dosage of mixed amphetamine salts-XR rechallenge, her symptoms of amotivation and poor concentration returned, although less severe. 

## 3. Discussion

Delusional parasitosis has been discussed in the literature, although there has not been a reported link between delusional parasitosis and mixed amphetamine salts. A recent article outlines other causal factors, yet the majority of recent publications are dedicated to the treatment of delusional parasitosis with antipsychotic medications.

In a 2010 report, Flann et al. [7] discusses three separate cases of delusional parasitosis in patients treated for Parkinson's disease. All three patients were taking dopamine agonists. Two patients were taking ropinirole, while the third was taking cabergoline. All patients reported abnormal beliefs of being infested with parasites, while one patient reported that the bubbles in a glass of water were insect larvae. After examination, there was no evidence of infestation among any of the patients. Upon discontinuation of the medications, delusions of all three patients resolved completely. 

In a 2006 article, Nicolato et al. [8] report treatment of ten patients with delusional parasitosis. The ten cases (seven females and three males) presented with differing dermatological signs of delusional parasitosis. All patients had a preexisting clinical comorbidity: four carrying a diabetes diagnosis, four with hypertension, and three with thyroid disease. None of the patients' clinical illnesses were linked to the delusional parasitosis. Upon examination, five patients were diagnosed with a delusional disorder that could not be linked to another psychiatric condition, two with major depressive disorder with psychotic features, two with dementia, and one carried a diagnosis of schizophrenia. All patients were treated with an anti-psychotic medication, both typical and atypical. These medications included haloperidol, pimozide, olanzapine, quetiapine, or risperidone. Six subjects achieved resolution of the delusional parasitosis completely, while three had partial improvement. In one case, no improvement was noted.

Kenchaiah et al. [9] extend the discussion regarding the use of atypical antipsychotics in the treatment of twenty individuals with delusional parasitosis. All patients described an infestation of parasites either on or inside their skin, while some experienced tactile hallucinations and even auditory hallucinations. Treatment usually involved the atypical antipsychotic risperidone or olanzapine. Haloperidol was used as were the antidepressants fluoxetine, sertraline, and imipramine. Although the anti-psychotic pimozide is traditionally associated with the treatment of delusional parasitosis, it was not used in this series. Among the twenty subjects, four maintained complete resolution of their delusional parasitosis. Eleven cases reported partial improvement, while the remainder were lost to followup.

Delusional parasitosis is an uncommon psychiatric condition. This case identifies the onset of symptoms with higher doses of stimulant medications. Discontinuation of mixed amphetamine salts led to rapid recovery and antipsychotic agents were not needed. Reintroduction of the medication at lower doses was successfully accomplished with therapeutic benefits again appreciated. Although this is a reportable case it must be emphasized that mixed amphetamine salts-induced delusional parasitosis is a rare occurrence. In our AD/HD specialty clinic, stimulant medications are prescribed regularly, often for patients that require doses that exceed FDA recommendations. This is done cautiously but generally without complications. Never before in our clinic had the onset of delusional parasitosis been identified. For this reason, other causes such as sleeplessness or marital discord cannot be excluded. Stimulant medications can play a vital role in patients suffering from ADHD and depression. As in all clinical decisions the potential benefits of treatment should be weighed against potential adverse events.
